# The role of lymph nodes in predicting the prognosis of ampullary carcinoma after curative resection

**DOI:** 10.1186/s12957-015-0643-1

**Published:** 2015-07-25

**Authors:** Shih-Chin Chen, Yi-Ming Shyr, Shu-Cheng Chou, Shin-E Wang

**Affiliations:** Division of General Surgery, Department of Surgery, Taipei Veterans General Hospital, National Yang Ming University, 10 F 201 Section 2 Shipai Road, Taipei, 112 Taiwan

**Keywords:** Ampullary carcinoma, Lymph node, Prognosis, Total harvested lymph node number

## Abstract

**Background:**

Lymph node involvement is one of the well-demonstrated prognostic factors in ampullary carcinoma. The aim of this study is to clarify the role of lymph nodes in predicting the survival outcome of ampullary carcinoma.

**Methods:**

A cohort of consecutive curative pancreaticoduodenectomies for ampullary carcinoma from 1999 to 2014 was retrospectively analyzed. The effect of node-associated variables, including lymph node status, positive lymph node number, total harvested lymph node (THLN) number, and lymph node ratio (LNR) was examined using univariate and multivariate analyses for survival outcome prediction.

**Results:**

In 194 evaluable patients, univariate analysis demonstrated that stage, cell differentiation, perineural invasion, and nodal status were significant conventional prognostic factors. Concerning the node-associated variables, positive nodal status, positive lymph node number ≥2, THLN number <14, and LNR ≥0.15 were significantly associated with poorer survival outcomes, with a 5-year survival rate of 20.3, 38.9, 25.4, and 18 %, respectively. By multivariate analysis, nodal status and THLN number were two independent predictors of survival. The most favorable 5-year survival rate was 84.4 % in patients with negative nodal involvement and THLN number ≥14, compared with the poorest 5-year survival rate of 16.1 % in those with positive nodal status and THLN number <14.

**Conclusions:**

Tumor biology reflected by lymph node status is the most important independent prognostic factor; nevertheless, surgical radicality based on THLN number also plays a significant role in the survival outcome for patients with ampullary carcinoma after curative pancreaticoduodenectomy.

## Background

Patients with periampullary carcinomas, consisting of pancreatic carcinoma, ampullary carcinoma, carcinoma of distal common bile duct, and duodenal carcinoma, have poorer prognosis compared with those with other gastrointestinal malignancies. Resection of periampullary carcinoma could provide a better survival outcome than non-operative biliary drainage or bypass operation [1]. For the proximity of anatomy, periampullary carcinomas share the same surgical strategy, which was known as pancreaticoduodenectomy (also called Whipple procedure [2]). Of periampullary carcinomas, the different biology of tumor origins could result in to some degree the difference of prognosis [3–6]. Ampullary carcinoma, one of the periampullary carcinomas, accounted for 0.2 % of all the gastrointestinal carcinomas and 6 % of periampullary tumors [7]. Patients with ampullary carcinomas had a favorable 5-year survival rate of >35 % after resection, compared with the worst 5-year survival rate of those with pancreatic carcinomas, around 7–20 % after resection [3–5, 8–12]. In addition, several clinicopathological factors, such as tumor size, resection margin, cell differentiation, and lymph node metastasis, have been comprehensively studied with respect to determining survival outcome after pancreaticoduodenectomy for ampullary carcinoma and other periampullary cancers [5, 12–14].

Of these clinicopathological prognostic factors, lymph node involvement was a well-demonstrated prognostic factor for ampullary carcinoma. However, the American Joint Committee on Cancer (AJCC) has classified the nodal status of ampullary carcinoma into only two categories, with or without regional lymph node metastasis [15]. Lately, many investigators tried to further define the importance of several aspects of lymph node involvement in various malignancies. The lymph node ratio (LNR), which was defined as the positive node number among the total harvested lymph node (THLN) number, attracted lots of attention. LNR was demonstrated to be a better prediction for survival outcome in many types of gastrointestinal malignancies including esophageal, gastric, pancreatic, colorectal, and of course ampullary carcinoma [16–29]. In addition, an increasing THLN number has also been claimed to result in more accurate staging and favorable survival outcome in many types of malignancies [25, 30–35]. Moreover, positive lymph node number, which represented the extension of a disease, was another established prognostic factor in various malignancies arising from the head and neck, breast, stomach, and colon and rectum, and it was well-categorized in these carcinomas with tumor-node-metastasis (TMN) staging system, but not for ampulla of Vater [15]. Regarding ampullary carcinoma, several authors had suggested the prognostic importance of positive lymph node number recently [36–39]. However, the significance of these node-associated variables on survival outcome is still not firmly established.

In light of these considerations, the aim of this study was to clarify the role of these node-associated factors in predicting the prognosis of ampullary cancer after curative resection.

## Methods

The data for patients with periampullary carcinomas who underwent pancreaticoduodenectomy between October 1999 and September 2014 were retrieved from a prospectively collected database for pancreatic surgery. Only patients with pathologic diagnosis of ampullary carcinoma, which was defined as malignancies arising from the anatomical structure of ampulla of Vater, were enrolled. Patients with the other periampullary malignancies, such as pancreatic carcinoma, carcinoma of duodenum, carcinoma of distal common bile duct, neuroendocrine neoplasm, intraductal papillary mucinous neoplasms, mucinous cystadenocarcinoma, acinar cell carcinoma, and solid pseudopapillary neoplasms, were excluded. Demographic characteristics and pathologic data were collected and analyzed.

All patients underwent a standard resection without extensive retroperitoneal lymph node dissection and pancreaticojejunostomy or pancreaticogastrostomy reconstruction. The territory of lymph node dissection consisted of resection of lymph nodes within the confined of the hepatoduodenal ligament, common hepatic artery, right side of the superior mesenteric artery, and inferior vena cava. Specimens were examined pathologically to determine the tumor size, cell differentiation, perineural invasion, lymphovascular invasion, residual tumor status, and lymph node status. Patients with incomplete lymph node record, undetermined cell differentiation, and diagnosis of carcinoma in situ were excluded from the analysis. Patients with in-hospital mortality were excluded as well since these patients might have the opportunity for better survival outcome after passing through the surgical events. Surgical resection with positive surgical margin, which was defined as the evidence of residual tumor at pancreatic neck and distal common bile duct cut-end, retroperitoneal margin, and superior mesenteric and portal vein grooves microscopically or grossly, was assumed as palliative resection. Patients with palliative resection were also excluded since there was a significant survival difference between the curative and palliative groups for periampullary carcinomas [40].

All continuous variables were presented as median (range) or as mean ± standard deviation (SD). When comparing the two groups, the continuous data were compared using Student’s *t* or Wilcoxon rank-sum test and the categorical variables were compared using chi-squared or Fisher’s exact test. We identified cutoff values of three node-associated variables by integrating the literature review with dividing the entire study population around the 75th percentiles. Actuarial survival was estimated using the Kaplan-Meier method, and univariate differences between the two subgroups were determined with a log-rank test. All the significant factors associated with lymph node in univariate analysis were added up into multivariate analysis subsequently. Cox proportional hazard ratio model was used in multivariate analysis to identify independent predictors of survival outcome. Hazard ratio (HR) and 95 % confidence intervals (CI) were calculated and reported. Statistics Version 21 (SPSS, Inc., Chicago, IL, USA) was used for all statistical analyses, and a *P* value of <0.05 was considered to indicate statistical significance.

## Results

**Fig. 1:**
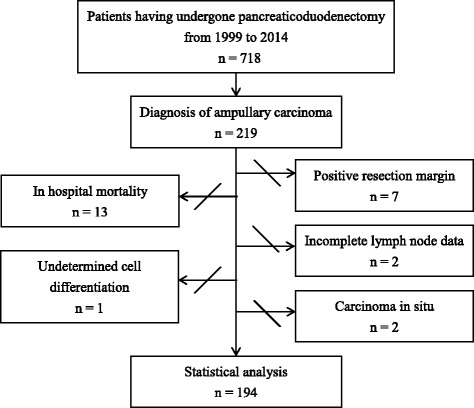
Inclusion and exclusion criteria for patients with ampullary carcinoma resulting in the final study cohort

During the study period, a total of 718 patients with periampullary cancers underwent pancreaticoduodenectomy surgery. Of these, 219 (30.5 %) patients were histologically confirmed with ampullary carcinoma. Exclusion criteria, as mentioned in the Methods section, were shown in-hospital mortality in 13 patients, palliative resection with positive margin in 7 patients, incomplete lymph node data in 2 patients, undetermined tumor differentiation in 1 patient, and carcinoma in situ in 1 patient (Fig. 1). Therefore, a total of 194 patients with ampullary carcinoma undergoing curative pancreaticoduodenectomy were included in the analysis.

There were 120 (61.9 %) men and 74 (38.1 %) women with a median age of 69 years (range 40–90), and the median tumor size was 2 cm (range 0.7–9.0 cm; 42.3 % ≥2 cm). Sixty-four patients (33 %) were stage I ampullary carcinoma, 110 patients (56.8 %) were stage II, and 20 patients (10.3 %) were stage III. On pathologic analysis, the majority of the tumors were moderate-differentiated (*n* = 137; 73.2 %), negative perineural invasion (*n* = 147; 75.8 %), and negative lymphovascular invasion (*n* = 153; 78.9 %). Seventy-two patients (37.1 %) had positive nodal disease, whereas 122 patients (62.9 %) had no nodal involvement. The 5-year survival rate of the entire cohort was 42.7 % with a median survival of 45.1 months and a mean of 83.2 ± 6.2 months. Of the conventional clinicopathological factors, univariate analysis demonstrated that stage, tumor differentiation, perineural invasion, and nodal status could significantly predict the prognosis for patients with ampullary carcinoma after curative resection. However, age, gender, tumor size, and lymphovascular invasion had no association with survival outcome (Table 1).

**Table 1:** Conventional prognostic factors for patients with ampullary carcinoma undergoing curative pancreaticoduodenectomy

Variables	No. of patients *n* (%)	Survival			*P* value
		5-year (%)	Median (months)	Mean ± SD (months)	
Age (years)					0.250
<60	57 (29.4)	49.0	57.7	88.5 ± 11.0	
≥60	137 (70.4)	40.7	41.8	78.9 ± 7.2	
Gender					0.121
Male	120 (61.9)	39.3	40.0	76.3 ± 8.1	
Female	74 (38.1)	48.2	57.7	93.8 ± 9.7	
Stage					<0.001
I	64 (33)	60.9	–	124 ± 11.0	
IIa	55 (28.4)	47.7	57.7	85.6 ± 10.9	
IIb	55 (28.4)	21.0	32.8	52.1 ± 9.4	
III	20 (10.3)	21.1	21.0	50.2 ± 13.9	
Tumor size (cm)					0.282
<2	112 (57.7)	46.0	54.4	88.3 ± 8.1	
≥2	82 (42.3)	37.8	40.0	74.3 ± 9.5	
Tumor differentiation					0.005
Well	22 (11.8)	42.3	45.1	76.7 ± 18.5	
Moderate	137 (73.2)	47.2	52.0	88.6 ± 7.1	
Poorly	28 (15)	25.4	23.0	31.7 ± 5.5	
Perineural invasion					0.045
No	147 (75.8)	48.5	54.4	89.8 ± 7.0	
Yes	47 (24.2)	20.1	34.1	58.3 ± 11.8	
Lymphovascular invasion					0.117
No	153 (78.9)	46.0	46.3	89.3 ± 7.0	
Yes	41 (21.1)	25.5	41.8	56.7 ± 10.6	
Nodal status					<0.001
Negative	122 (62.9)	53.9	79.5	98.5 ± 7.9	
Positive	72 (37.1)	20.3	28.5	53.4 ± 8.6	

*SD* standard deviation

Table 2 demonstrated the roles of node-associated factors in predicting survival outcome for patients with ampullary carcinoma undergoing curative pancreaticoduodenectomy. The univariate analysis revealed that nodal status, positive lymph node number, THLN number, and LNR were all significantly associated with survival outcome. These significant node-associated variables were enrolled subsequently in multivariate Cox proportional hazard regression analysis, which revealed that nodal status and THLN number were independent prognostic factors, whereas positive lymph node number and LNR failed to do so. Patients with positive nodal status had an increased risk of death (HR = 2.12, 95 % CI, 1.05–4.24; *P* = 0.036) compared with those with negative nodal status. Patients with THLN number ≥14 had improved survival outcome (HR = 0.49, 95 % CI, 0.26–0.93; *P* = 0.028) in contrast with patients with THLN number <14.

**Table 2:** The roles of node-associated factors in predicting overall survival outcome for patients with ampullary carcinoma undergoing curative pancreaticoduodenectomy

Variables	No. of patients *n* (%)	Survival			Univariate analysis	Multivariate analysis		
		5-year (%)	Median (months)	Mean ± SD (months)	*P* value	Hazard ratio	95 % CI	*P* value
Nodal status					<0.001			0.036
Negative	122 (62.9)	53.9	79.5	98.5 ± 7.9		(Ref.)	–	
Positive	72 (37.1)	20.3	28.5	53.4 ± 8.6		2.12	1.05–4.26	
THLN number					0.037			0.028
<14	148 (76.3)	38.9	41.8	73.9 ± 6.9		(Ref.)	–	
≥14	46 (23.7)	56.9	–	109.9 ± 13.5		0.49	0.26–0.93	
Positive LN number					0.004			0.938
<2	150 (77.3)	47.2	54.4	90.3 ± 7.2		(Ref.)	–	
≥2	44 (22.7)	25.4	25.7	58.3 ± 11.5		1.03	0.50–2.14	
LNR					<0.001			0.665
<0.15	147 (75.8)	50.3	71.8	94.6 ± 7.3		(Ref.)	–	
≥0.15	47 (24.2)	18.0	26.2	46.4 ± 8.6		1.20	0.53–2.75	

*THLN* total harvested lymph node, *LN* lymph node, *LNR* lymph node ratio, *SD* standard deviation, *CI* confidence interval

Clinicopathological characteristics of the cohort categorized by lymph node status were listed in Table 3. There was no statistical difference between patients with positive lymph node status and negative lymph node status in terms of age and gender. More patients with lymph node involvement had higher stage, had a larger tumor size, and had higher rates of poorly differentiated tumors compared with those without lymph node involvement. Also, the presence of lymphovascular invasion and perineural invasion was significantly higher in patients with positive lymph node status (all *P* < 0.05).

**Table 3:** Demographics of patients categorized with lymph node status

Variables	Negative nodal status	Positive nodal status	*P* value
	*n* = 122	*n* = 72	
Age (years)			0.545
Median	69	69.5	
Range	40–90	40–90	
Mean ± SD	66.7 ± 11.7	67.8 ± 10.8	
Gender			0.880
Male	76 (62.3 %)	44 (61.1 %)	
Female	46 (37.7 %)	28 (38.9 %)	
Stage			<0.001
I	64 (52.5 %)	0	
IIa	55 (45.1 %)	0	
IIb	0	55 (76.4 %)	
III	3 (2.4 %)	17 (23.6 %)	
Tumor size			0.007
Median	1.9	2.4	
Range	0.7–7.0	0.8–9.0	
Mean ± SD	2.2 ± 1.2	2.7 ± 1.5	
Tumor differentiation			0.002
Well	20 (17.1 %)	2 (2.9 %)	
Moderate	85 (72.6 %)	52 (74.3 %)	
Poorly	12 (10.3 %)	16 (22.9 %)	
Lymphovascular invasion			<0.001
No	109 (89.3 %)	44 (61.1 %)	
Yes	13 (10.7 %)	28 (38.9 %)	
Perineural invasion			<0.001
No	104 (85.2 %)	43 (69.7 %)	
Yes	18 (14.8 %)	29 (40.3 %)	

*SD* standard deviation

**Fig. 2:**
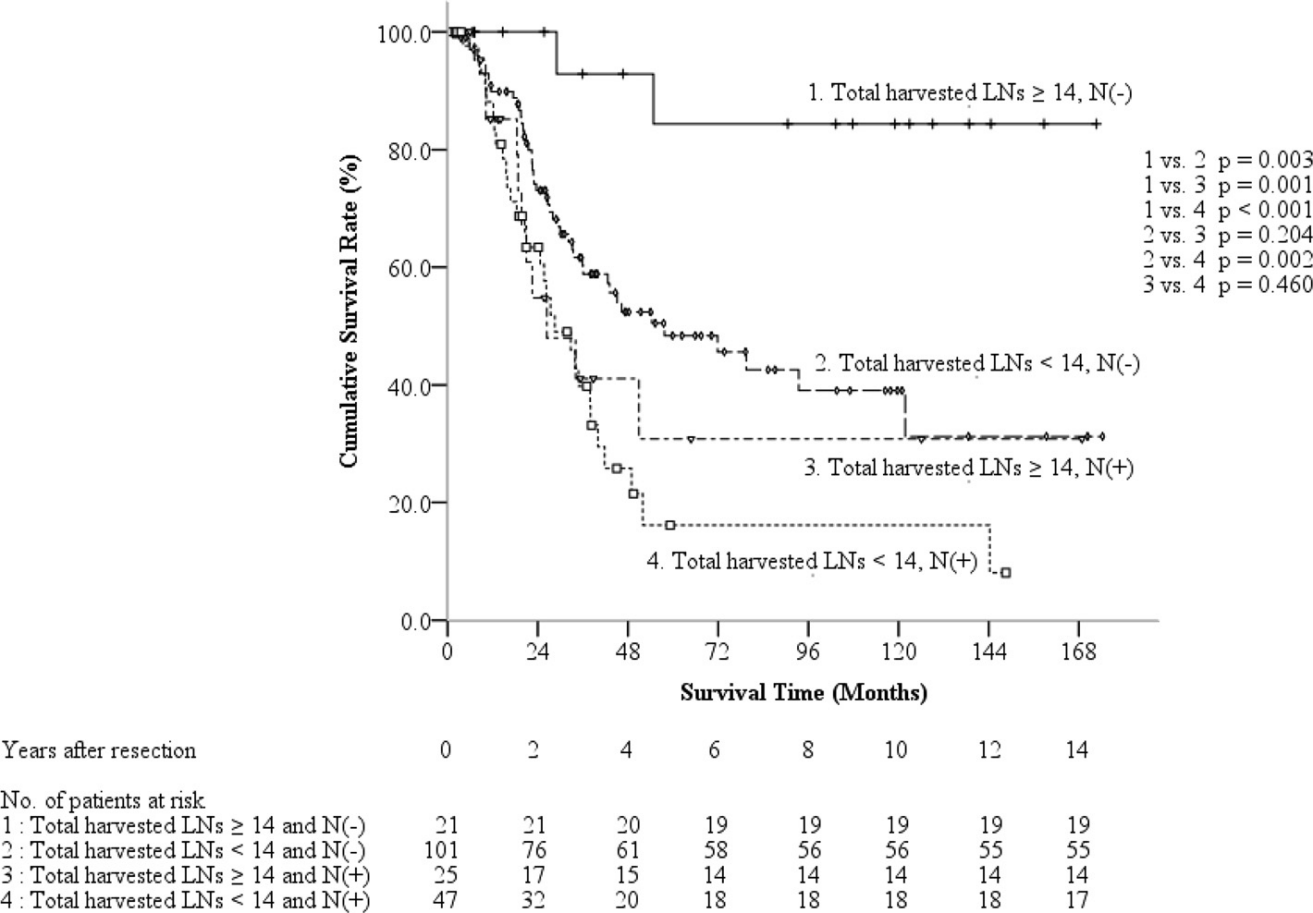
Comparison of survivals for patients with ampullary carcinoma after curative pancreaticoduodenectomy based on nodal status and total harvested lymph node number

In the following survival analysis, patients were stratified into four subgroups based on the independent predictors of nodal status and THLN number (Fig. 2). Patients with negative nodal status and THLN number ≥14 had the most favorable 5-year survival rate of 84.4 %, followed by those with negative nodal status and THLN number <14 (5-year survival rate 48.3 %) and those with positive nodal status and THLN number ≥14 (30.8 %). The lowest 5-year survival rate was found in patients with positive nodal status and THLN number <14 (16.1 %). In the node-negative subgroups, patients with THLN number ≥14 had significant improved survival outcome compared with those with THLN number <14 (*P* = 0.003). However, in the node-positive subgroups, the survival rate was not statistically different though patients with THLN number ≥14 had a higher 5-year survival rate (*P* = 0.46). There was no survival difference between patients with negative nodal status and THLN number <14 and those with positive nodal status and THLN number ≥14 (*P* = 0.204).

## Discussion

The prognostic significance of lymph node involvement in cancers is well-established in tumor-node-metastasis system of AJCC [15]. Of all the other malignancies except ampullary carcinoma, lymph node disease is often further categorized into several groups based on either positive lymph node number or location. However, nodal status of ampullary carcinoma is only classified simply into only two categories, with or without regional lymph node metastasis. Recently, many authors claimed that several node-associated factors, including positive lymph node number and LNR, could have the improved efficacy for prognostic prediction in ampullary carcinoma [6, 24–26, 36–39]. Indeed, the current study demonstrated that lymph node status and THLN number, rather than positive lymph node number or LNR, independently determined survival outcome of patients with ampullary carcinoma after curative resection. The best survival outcome was seen in patients with negative nodal status and THLN ≥14 with a favorable 5-year survival rate of 84.4 %, which was much better than that of patients in other categories. The importance of these two factors in ampullary carcinoma had been shown in studies separately based on the smaller population [9, 25, 30, 41]. The present study emerged that both lymph node status and THLN number were independent prognostic factors of ampullary carcinoma from a large single-institute data.

Lymph node involvement might imply that the tumor behavior changes on its way from confinement in origin organ toward distant metastasis. This aggressiveness of dissemination via lymphatic system could result in poorer survival outcome. This study revealed that 32 patients (37.1 %) with positive nodal status had poorer survival outcome with a 5-year survival rate of 20.3 %, compared with 53.9 % in those with negative nodal disease. By multivariate analysis, nodal status becomes one of the independent prognostic factors. In our study cohort, patients with positive lymph node status had significant higher stage, larger tumor size, higher grade of cell differentiation, presence of perineural invasion, and lymphovascular invasion (all *P* < 0.05, Table 3). All these findings indicated that tumors in patients with positive nodal status have the tendency toward poorer prognosis. Therefore, nodal status seems to be a reflection of tumor biology.

Although positive lymph node number and LNR were significant prognostic factors by univariate analysis in this study, these two factors failed to become independent factors by multivariate analysis. Meanwhile, some studies held the opposite conclusions [24, 25, 28, 36–39]. Sakata et al. [38] examined 71 patients with ampullary carcinoma undergoing pancreaticoduodenectomy with regional lymphadenectomy and 34 patients had lymph node metastasis. The positive lymph node number was identified as an independent prognostic factor in multivariate analysis. The cumulative 5-year survival rate was 63 % for patients with 1–3 positive node whereas 0 % for those with ≥4 positive nodes. LNR, another node-associated factor, has drawn much attention for predicting the survival outcome in patients with various gastrointestinal malignancies [16, 17, 19–21]. LNR may to some degree compensate or standardize variations in inadequate surgical lymph node dissection [27]. However, some studies proved that LNR could become an independent prognostic factor in pancreatic carcinoma, instead of ampullary carcinoma [23, 26, 28, 29]. Recently, Pomianowska et al*.* compared the three periampullary carcinomas and demonstrated that LNR may be a powerful prognostic factor only in pancreatic carcinoma. Lymph node status adequately determined the prognosis in ampullary carcinoma [9].

The THLN number might indicate the quality and radicality of surgical lymphadenectomy. Evaluation of limited THLNs could bring a major impact on survival outcome resulting in underestimation of staging and prognosis in various malignancies [25, 32, 42]. Smith et al*.* demonstrated that the more the THLN number examined, the better the resulting postgastrectomy survival rate in gastric cancer. A linear trend for superior survival based on more THLNs could be confirmed for all four stage subgroups of gastric cancer. A cut-point analysis yielded the greatest survival difference at ten THLNs [32]. Le Voyer et al*.* also reported that THLN number was a significant variable affecting survival outcome among 3411 colorectal cancer patients [42]. In ampullary carcinoma, the evidence of increasing THLN number resulting in better prognostic determination was also observed in AJCC. Using the THLN number 12 to stage the disease properly was recommended in the 7th edition of AJCC [15]. Our study demonstrated that patients with THLN number ≥14 had a better 5-year survival rate of 56.9 % compared with that of 38.9 % in patients with THLN number <14 (*P* = 0.037). Furthermore, THLN number independently affected the survival outcome. The result is echoed by Falconi et al*.* in a study of 90 patients with ampullary carcinoma after curative resection, which suggested that adequate THLN number of 16 had favorable survival outcome [25]. Adequate lymph node dissection based on larger THLN number could offer more “correct” lymph node count or more “accurate” staging. Smith et al. proposed the mechanism “stage migration” that patients with larger THLN number had superior survival outcome in gastric malignancy [32]. In their study, statistics of linear regressions revealed a proportional increase in TNM stage as the THLN number increased. Slidell et al. also suggested that patients without lymph node involvement who had fewer than 12 THLNs might be understaged [34]. Therefore, THLN number which could be a function of surgical radicality or quality plays a significant role in prognosis and accurate staging for ampullary carcinoma. The evidence of these node-associated variables on survival outcome is still not firmly established. Several studies reported the impact of THLN on the outcomes of ampullary carcinoma based on relative smaller population. To the best of our knowledge, the current cohort demonstrates the conclusion based on the largest population.

Since THLN number and nodal status were both independent prognostic factors of ampullary carcinoma, as expected, patient with THLN number ≥14 and negative nodal status had the most favorable survival outcome in this study. In contrast, patients with THLN number <14 and positive nodal status had poorest survival outcome. Our analysis also revealed that patients with THLN number ≥14 had better survival outcome compared with those with THLN number <14 among node-positive subgroups (5-year survival rate 30.8 vs. 16.1 %). Furthermore, there was no significant survival difference between patients with THLN number <14 and negative nodal status and those with THLN number ≥14 and positive nodal status. These findings indicate that adequate lymph node dissection of THLN number ≥14 might compensate the survival difference caused by adverse tumor biology in patients with lymph node involvement. In addition, the result that patients with THLN number <14 had poorer survival rate than those with THLN number ≥14 among node-negative subgroups might imply that inadequate lymph node dissection might compromise the survival outcome with favorable tumor biology. These findings could also provide further evidences to support that these two factors, nodal status and THLN number, affect the prognosis independently.

## Conclusions

Lymph node status which could indicate tumor biology is the most powerful independent prognostic factor for patients with ampullary carcinoma undergoing curative resection. Nevertheless, THLN number is also an independent prognostic factor. Patients with better tumor biology of negative nodal status and adequate surgical radicality of THLN number ≥14 have the most favorable survival outcome. The effort of lymph node dissection based on THLN number could compensate or compromise the prognostic difference caused by tumor biology. However, either surgical radicality or stage migration might be the possible reason to explain why THLN number impacts on survival outcome, which needs to be clarified by further studies.

## Abbreviations

AJCC: American Joint Committee on Cancer
CI: confidence interval
HR: hazard ratio
LNR: lymph node ratio
SD: standard deviation
THLN: total harvested lymph node
